# Driving habits and behaviors of patients with brain tumors: a self-report, cognitive and driving simulation study

**DOI:** 10.1038/s41598-018-22937-y

**Published:** 2018-03-15

**Authors:** Ann Mansur, Megan A. Hird, Alexa Desimone, Iryna Pshonyak, Tom A. Schweizer, Sunit Das

**Affiliations:** 1 University of Toronto Faculty of Medicine, Toronto, Ontario Canada; 2 Li Ka Shing Knowledge Institute, St. Michael’s Hospital, Toronto, Ontario Canada; 3 Institute of Biomaterials and Biomedical Engineering, University of Toronto, Toronto, Ontario Canada; 4 Division of Neurosurgery, St. Michael’s Hospital, Toronto, Ontario Canada

## Abstract

The purpose of the study is to determine driving habits and behaviors of patients with brain tumors in order to better inform discussions around driving safety in this population. Eight-four patients with brain tumors participated in a survey on their driving behaviors since their diagnosis. Thirteen of these patients and thirteen sex- and age-matched healthy controls participated in cognitive testing and several driving simulation scenarios in order to objectively assess driving performance. Survey responses demonstrated that patients with brain tumors engage in a variety of driving scenarios with little subjectve difficulty. On the driving simulation tasks, patients and healthy controls performed similarly except that patients had more speed exceedances (U = 41, p < 0.05) and a greater variability in speed (U = 57, p < 0.05). Performance on the selective attention component of the UFOV was significantly associated with greater total errors in the Bus Following task for patients with brain tumors compared to healthy controls (rs = 0.722, p < 0.05, CI [0.080, 0.957]). Better comprehensive driving assessments are needed to identify patients with driving behaviors that put themselves and others at risk on the road.

## Introduction

Driving is a complex task that requires the integration of motor skills, visual-spatial tracking, and higher-order executive skills. Many of these functions are compromised in patients with brain tumors, either by direct disruption of eloquent parenchyma, associated changes in the brain such as increased intracranial pressure and edema, seizure activity, or treatment effects.

Determining fitness to drive is a moral and often legal responsibility of physicians around the world. Physician guidelines established by the Canadian Medical Association^1^, American Medical Association^2^, Austroads and the National Transport Commission of Australia^3^, and the UK Driver and Vehicle Licensing Agency^4^, state that patients should not drive if their medical condition or treatments impair their driving safety. For patients with brain tumors, Canadian governing bodies have dictated cessation of driving for 6 months following the last seizure if they have well-controlled epilepsy, and for 12 months if they have a seizure after surgery^1^. These governing bodies have been less direct in offering guidelines for physicians in their assessment of driver safety and fitness to drive.

The lack of established best practices in this population stems in part from a lack of empirical research on the driving performance of patients with brain tumors, especially those with resected brain tumors who are either seizure-free or past the 12-month seizure-free period. Current driving guidelines do not take into account the driving habits and performance of patients with brain tumors, and it remains unclear as to which patients should be deemed unfit to drive. The aim of this study was to identify the self-reported driving habits and behaviors of patients with a variety of brain tumor diagnoses and to use simulator technology to characterize the driving performance of patients with brain tumors against that of healthy age- and gender-matched controls. A secondary aim was to identify if certain cognitive tasks correlated with deficits in driving performance.

## Materials and Methods

This study was conducted at the Li Ka Shing Knowledge Institute in Toronto, Canada, and was approved by the Research Ethics Board at St. Michael’s Hospital (REB 16-102). The methods of this study were conducted in accordance with this board’s regulations. All participants provided written informed consent prior to their inclusion in the study. The datasets generated or analyzed during the current study are available from the corresponding author on reasonable request.

### Part 1: Self-report questionnaires

#### Participants

Adult patients at St. Michael’s Hospital between the ages of 18 and 80 with a diagnosis of a brain tumor, who were proficient in English (in order to read and complete the survey), had a valid driver’s license (Full G license certified in Ontario, which also reflects visual acuity in accordance with driving regulations), and did not have any other neurological or psychiatric illness, were considered for the study. Eligible participants were identified by the senior author (S.D.). Two hundred and nine patients were screened for the study, of which 60 did not drive or had no license, 39 were lost to follow-up, 5 were unable to participate because of language or visual barriers, and 19 declined. Of the 105 eligible participants, 86 (81.9% response rate) underwent surveys of their driving habits and behaviors.

#### Procedures

Participants completed two self-report surveys of their driving habits and behaviors. The first was a survey of their driving habits since their brain tumor diagnosis, which was adapted from the Driving Habits Questionnaire (DHQ) by Owsley and colleagues^5^ (see Appendix A). The second was the American Academy of Neurology Patient Questionnaire^6^.

### Part 2: Driving simulation and cognitive testing

#### Participants

Adult patients at St. Michael’s Hospital between the ages of 18 and 80 with a diagnosis of a brain tumor, who were proficient in English (in order to read and complete the survey), had a valid driver’s license (Full G license certified in Ontario), driven at least once since their diagnosis, had normal to corrected vision, were seizure-free, and did not have any other neurological or psychiatric conditions, were considered. Twenty-six participants were included in this part of the study (brain tumor, n = 13; healthy controls, n = 13). All patients underwent transcranial or endoscopic transsphenoidal brain tumor resection at St. Michael’s Hospital by the senior author (S.D.).

Healthy control participants were volunteers recruited from the community and had no prior history of neurological or psychiatric illness. Volunteers were between the ages of 18 and 80, proficient in English, and had a valid Full G driver’s license certified in Ontario. Healthy control participants were age- and sex-matched to the brain tumor participants.

#### Procedures

Driving simulation. A portable single-screen driving simulator (Logitech G25 model, STISIM Drive®), which included a steering wheel, accelerator pedal, brake pedal, and signalling system, was used to assess driving performance^7^. All participants completed one training scenario and two experimental sessions – the City Driving scenario and Bus Following task^7^. The City Driving scenario involves conditions that vary in complexity, including straight driving, right and left turns, and left turns with oncoming traffic. Left turns with oncoming traffic require greater cognitive demand, as participants need to judge when it is safe to turn^7,8^. In the Bus Following task, participants need to follow a lead vehicle that continuously varies its speed while maintaining a safe and consistent distance from the vehicle. Successful completion of this task requires sustained attention, error-monitoring, and visual-motor control^9^.

Variables of interest included: collisions, speed limit exceedance, centreline crossings, road edge excursions, “Stop” signs missed (City Driving scenario only), total driving errors (the sum of individual errors listed above), turning errors (City Driving scenario only; sum of collisions, centreline crossings, and road edge excursions across right turns, left turns, and left turns with traffic), percentage time out of the legal driving lane and over the posted speed limit, variability in lane position (standard deviation in lane positioning, SDLP), and variability in speed (standard deviation in speed). Errors were analyzed separately for the City Driving and Bus Following scenarios.

Cognitive testing. After the driving simulation, participants completed a cognitive battery including^1^: measures of frontal (executive) function, such as the Trails-Making Test Parts A and B, Digit Span, Digit Symbol, and a test of verbal semantic and fluency^2^; the Hospital Anxiety and Depression score for mood; and^3^ the Useful Field of View (UFOV), which is a measure of vision and visual attention that requires participants to rapidly detect, locate, and identify a target embedded in a complex environment^10^. For healthy controls, the Montreal Cognitive Assessment (MoCA) was added to this battery as a screening of overall global cognitive functioning. All healthy control participants included in the study scored ≥ 26 on the MoCA.

### Statistical analyses

Cognitive test scores and driving simulator data collected from patients with brain tumors and healthy controls were compared using the non-parametric Mann-Whitney U test. Secondary correlation analyses investigating the association between driving performance and cognitive scores for patients with brain tumors were conducted using the Spearman’s rank correlation with bootstrapping.

## Results

### Part 1: Self-report questionnaires

**Figure 1:**
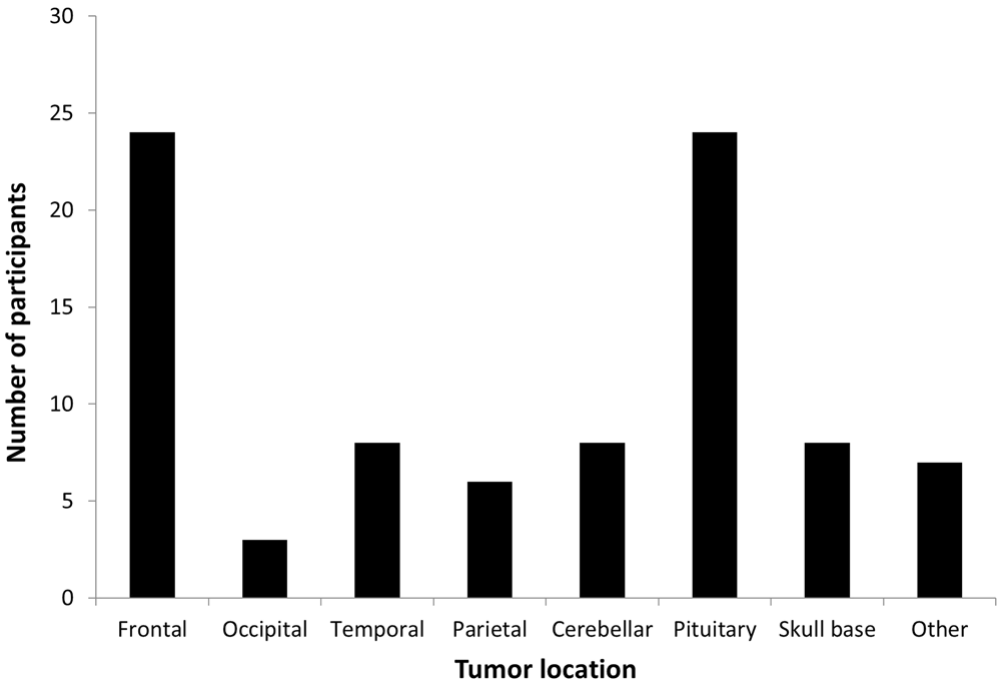
Tumor location. Bar graph data values: Frontal: 24 (27.3%). Occipital: 3 (3.4%). Temporal: 8 (9.1%). Parietal: 6 (6.8%). Cerebellar: 8 (9.1%). Pituitary: 24 (27.3%). Skull base: 8 (9.1%). Other: 7 (8.0%).

Demographic and clinical data of the patients included in this study are presented in Tables 1 and 2, respectively. The average age of the patients was 54.6 years and the average time since diagnosis was 56.5 months. Most patients had a meningioma, pituitary adenoma, or glioma. Almost three-quarters (70.9%) of patients had their tumors resected; the remaining patients underwent biopsy or were observed with serial imaging. The most common tumor locations were in the frontal lobe and sella (Fig. 1).

**Table 1:** Demographics of the brain tumor participants.

	Participants (N = 86)
Age	54.6 (14.1)
Gender, male (N, %)	44 (51.2)
Presenting complaint	
Seizures	15 (17.4)
Headache	30 (34.9)
Nausea/vomiting	4 (4.7)
Vision	20 (23.3)
Speech disturbance	3 (3.5)
Gait disturbance	4 (4.7)
Syncope	5 (5.8)
Vertigo	5 (5.8)
Sensory changes*	6 (7.0)
Asymptomatic	6 (7.0)
Time since diagnosis,	56.5 (54.3)
months	54.9 (58.5)
Time since surgery, months	
Tumor size (cm^3^)	36.3 (66.1)

Values are reported in mean (standard deviation) format unless otherwise specified. N = number of participants.

*Sensory changes include numbness, tingling and pain.

**Table 2:** Clinical characteristics of the brain tumor participants.

	Participants (N, %)
Location (N = 86)	
Frontal*	24 (27.9)
Occipital	3 (3.5)
Temporal	8 (9.3)
Parietal	6 (7.0)
Cerebellar	8 (9.3)
Pituitary	24 (27.9)
Skull base	8 (9.3)
Other**	9 (10.5)
Side of tumor (N = 86)	
Right	28 (32.6)
Left	27 (31.4)
Bilateral	3 (3.5)
Midline	28 (32.6)
Tumor grade (N = 41)	
Low grade	34 (82.9)
High grade	7 (17.1)
Histology (N = 58)	
Glioma	14 (24.1)
Pituitary adenoma	18 (31.0)
Meningioma	18 (31.0)
Craniopharyngioma	1 (1.7)
Hemangioblastoma	2 (3.4)
Medulloblastoma	1 (1.7)
Gangliocytoma	2 (3.4)
Schwannoma	2 (3.4)
Treatments (N = 86)	
Surgery	61 (70.9)
Chemotherapy	8 (9.3)
Radiotherapy	7 (8.1)

Values are reported in mean ± standard deviation format unless otherwise specified. N = number of participants.

*Two patients with frontotemporal tumors (counted as frontal and temporal) and one patient with a frontoparietal tumor (counted as frontal and parietal).

**Other: tumors in the ventricular system, basal ganglia, tentorium, falx.

Self-reported driving habits and behaviors are reported in Table 3. The average years of driving experience was 31.6 years and the average number of hours driving per week was 11. No patients self-reported their driving quality as “poor” or “fair”. Over half reported their driving quality as “good” (52.3%) and over a third reported it as “excellent” (36%). The majority of patients preferred to drive themselves if they needed transportation. Approximately 40% of patients had at least one collision since their diagnosis.

**Table 3:** Self-reported driving habits and behaviours of patients with brain tumors since diagnosis.

Driving habits	Participants (N = 86)	
Driving experience, years	31.6 (13.6)	
Driving experience, hours/week	11.0 (13.2)	
Self-reported quality of driving (N,%)		
Poor	0(0)	
Fair	0(0)	
Average	10 (11.6)	
Good	45 (52.3)	
Excellent	31 (36.0)	
Preferred method of transportation (N, %)		
Public transport	6 (7.0)	
Someone else	8 (9.3)	
Drive self	66 (76.7)	
Bicycle	3 (3.5)	
Walk	3 (3.5)	
Collisions (N, %)	34 (39.5)	
**Driving behaviours**	**Participants (N = 84)**	Difficulty* (mean, SD)
Driving in rain/snow	82 (97.6)	3.4 (0.7)
Left-hand turns in on-coming traffic	81 (96.4)	3.8 (0.5)
Driving on highway	79 (94.0)	3.8 (0.5)
Driving in rush-hour traffic	74 (88.1)	3.8 (0.5)
Driving at night	80 (95.2)	3.6 (0.6)
Driving faster than speed limit if won’t get caught**	14 (16.7)	
Will run a red light if thinks he/she will not get caught**	3 (3.6)	
Has concerns about driving ability**	1 (1.2)	
Others have concerns about their driving ability**	3 (3.6)	
Angry behaviour to other drivers**	8 (9.5)	
Limited the amount of driving since diagnosis** (N, %)	15 (17.9)	

Values are reported in mean ± standard deviation format unless otherwise specified. N = number of participants.

*Difficulty was rated between a 1 to 5 scale, where 1 was extremely difficult and 5 was not difficult at all.

**Incidence reported here is taken from positive responses (ie. “agree” or “strongly agree”).

In terms of driving behaviors, almost all patients reported that they drive during rainy or snowy conditions (97.6%), at night (95.2%), and on the highway (94%), with little self-identified difficulty. Fourteen patients (16.7%) indicated that they drive faster than the speed limit if they think they won’t get caught and eight (9.5%) will yell, honk or make gestures at other drivers if they get mad while driving. Only one (1.2%) participant reported concerns with their own driving behaviors. Lastly, almost 20% of participants indicated that they limited their amount of driving since their brain tumor diagnosis.

**Figure 2:**
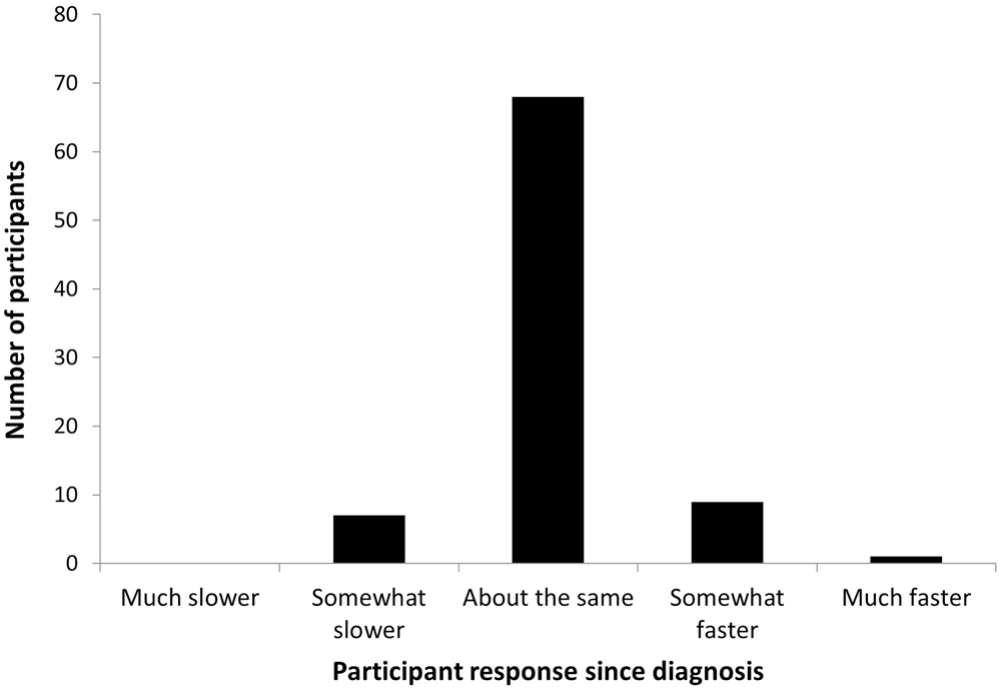
Driving speed compared to the general flow of traffic in the city. Bar graph data values: Much slower: 0 (0%). Somewhat slower: 7 (8.1%). About the same: 68 (79.1%). Somewhat faster: 9 (10.5%). Much faster: 1 (1.2%).

**Figure 3:**
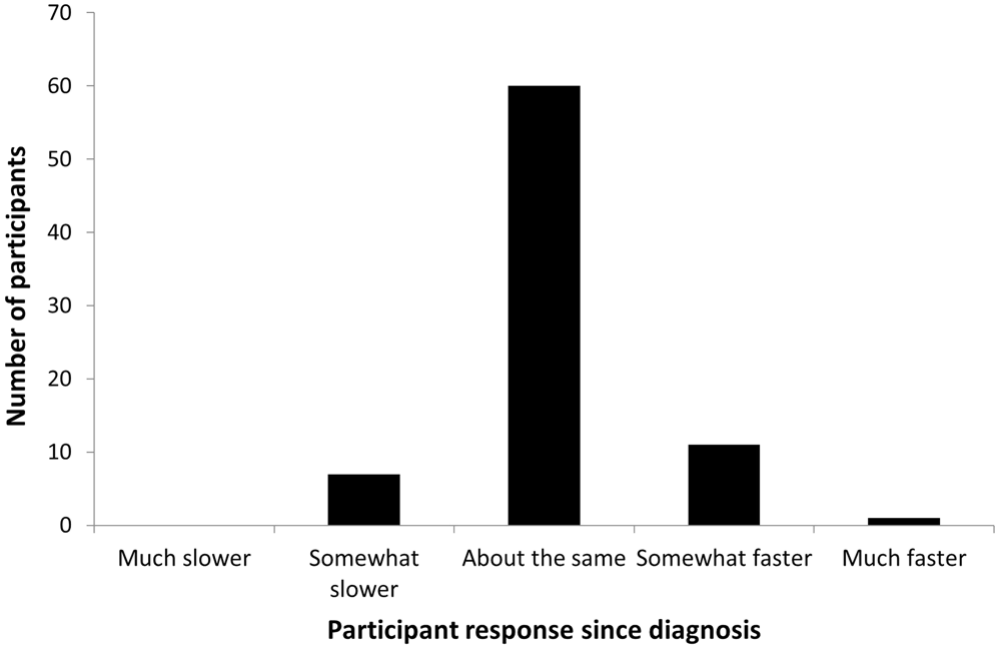
Driving speed compared to the general flow of traffic on the highway. Bar graph data values: Much slower: 0 (0%). Somewhat slower: 7 (8.9%). About the same: 60 (75.9%). Somewhat faster: 11 (13.9%). Much faster: 1 (1.3%).

Patient self-reported driving speed compared to the general flow of traffic in the city and on the highway are shown in Figs 2 and 3, respectively. Over three-quarters of patients responded that their driving speed is about the same as the general flow of traffic in both city (79.1%) and highway (75.9%) driving. Only one participant indicated that they drive much faster than the general public in both driving environments. No patients indicated that they drive slower than the general flow of traffic.

### Part 2: Driving simulation and cognitive testing

Demographic and cognitive scores of patients and controls are presented in Table 4. Clinical data for this cohort of patients is presented in the Supplementary Data. Patients and healthy controls performed comparably well on cognitive testing of frontal lobe function (TMT-A, TMT-B, UFOV); however, patients reported having more emotional deficits than healthy controls, with a higher HADS Anxiety score (U = 30, p < 0.05) and overall HADS score (HADS Anxiety + HADS Depression; U = 30, p < 0.05).

**Table 4:** Demographic and cognitive scores of participants with brain tumor and healthy age- and sex-matched control.

	Healthy Controls (n = 13)	Brain Tumour Participants (n = 13)	p-value
Age	49.8 (13.9)	49.0 (13.8)	0.880
Gender, male N (%)	7 (53.8%)	7 (53.8%)	1.00
Driving experience, years	31.0 (14.2)	30.1 (16.5)	0.810
Driving experience, hours/week	5.4 (5.9)	11.0 (10.4)	0.079
Self-reported accidents	0.8 (1.0)	0.7 (0.7)	0.960
TMT-A time	19.7 (4.7)	24.5 (10.0)	0.228
TMT-A errors	0(0)	0.1 (0.3)	0.776
TMT-B time	43.8 (18.4)	55.1 (24.5)	0.228
TMT-B errors	0.5 (0.9)	0.3 (0.6)	0.608
UFOV Processing Speed	19.0 (5.0)	24.7 (19.0)	0.976
UFOV Divided Attention	25.0 (11.3)	54.4 (71.4)	0.797
UFOV Selective Attention	108.5 (30.7)	82.4 (36.3)	0.152
HADS Anxiety	3.7 (2.4)	6.8 (3.0)	**0.030**
HADS Depression	1.6 (1.9)	3.5 (2.6)	0.057
HADS total score	4.8 (3.5)	10.2 (5.1)	**0.015**

Values are reported in mean ± standard deviation format unless otherwise specified. N, number of participants; HADS, Hospital Anxiety and Depression Scale; TMT, Trail Making Test; UFOV, Useful Field of View; %, percentage. p-values reported for non-parametric Mann-Whitney U test.

Driving simulator data are reported in Table 5. On average, brain tumor patients had significantly greater variability in speed than controls during the City Driving scenario (U = 57, p < 0.05), and they committed more than twice the number of speed exceedances as controls during the Bus Following task (U = 41, p < 0.05). Brain tumor patients and healthy controls did not differ significantly on number of collisions, turning errors, or total errors committed in both driving scenarios.

**Table 5:** Driving simulator results across participants with brain tumor and healthy control participants across the City Driving scenario and the Bus Following task.

	Healthy Controls (n = 13)	Brain Tumour (n = 13)	p-value
*City Driving Scenario*			
Length of run	1337.5 (190.85)	1255.5 (92.4)	0.336
Collisions	0.3 (0.6)	0.4 (0.8)	0.960
Speed exceedances	8.8 (6.8)	12.8 (6.4)	0.125
Centreline crossings	1.6 (0.6)	3.7 (4.8)	0.511
Road edge excursions	0.4 (0.2)	0.2 (0.6)	0.762
Stop signs missed	1.4 (0.3)	1.7 (1.6)	0.840
Total errors*	12.5 (8.2)	18.8 (10.3)	0.091
% time out of legal driving lane	0.3 (0.5)	0.6 (0.7)	0.650
% time over speed limit	3.1 (3.1)	4.6 (3.8)	0.169
SDLP	4.4 (0.2)	4.3 (0.2)	0.139
SD in speed	20.4 (1.7)	21.9 (1.4)	**0.029**
Right turn errors	0.9 (2.2)	1.1 (1.8)	0.545
Left turn errors^†^	0.1 (0.4)	0(0)	0.511
Left turn with traffic errors^†^	0.7 (0.6)	0.8 (0.8)	0.920
Total turning errors^†^	1.8 (2.3)	1.9 (2.4)	0.960
*Bus Following Task*			
Length of run	287.0 (11.7)	289.8 (11.2)	0.687
SD range from bus	17.3 (9.0)	18.5 (10.6)	0.762
Speed exceedances	0.8 (1.2)	1.8 (1.3)	**0.026**
Centreline crossings	(0.4)	0.7 (1.5)	0.687
Road edge excursions	2.3 (4.0)	0.8 (1.7)	0.511
Total errors^‡^	3.2 (5.0)	3.2 (3.2)	0.390
% time out of legal driving lane	1.6 (2.7)	0.7 (1.5)	0.724
% time over speed limit	0.8 (1.5)	1.7 (2.6)	0.072
SDLP	0.3 (0.1)	0.3 (0.1)	0.960
SD in speed	8.2 (1.6)	9.3 (2.5)	0.125

All values reported in mean ± standard deviation format. ^%^Percentage of. SD, standard deviation. SDLP, standard deviation in lane position. *City Driving total errors are the sum of: collisions, speed exceedances, centerline crossings, road edge excursions, stop signs missed. ^†^Turning errors are the sum of collisions, centerline crossings, and road edge excursions committed during right turns, left turns, left turns with oncoming traffic, and all turn types (sum of right + left + left turn with traffic errors). ^‡^Bus Following total errors is the sum of: speed exceedances, centerline crossings, road edge excursions. p-values reported for non-parametric Mann-Whitney U test.

Correlation results between driving simulator errors, clinical behaviors, self-reported data and cognitive tests scores for patients are reported in Table 6. Greater time since diagnosis was positively correlated with an increased number of total errors in City Driving (*r*_*s*_ = 0.798, p < 0.05, CI [0.462, 0.950]) and Bus Following (*r*_*s*_ = 0.840, p < 0.01, CI [0.534, 0.951]), and a greater number of speed exceedances in Bus Following (*r*_*s*_ = 0.753, p < 0.05, CI [0.357, 0.908]). Patients who reported avoiding driving in busy traffic tended to have fewer speed exceedances in City Driving (*r*_*s*_ = −0.658, p < 0.05, CI [−0.817, −0.296]). Performance on the selective attention component of the UFOV (i.e. with a higher score indicating greater impairment) was significantly associated with greater total errors in the Bus Following task (*r*_*s*_ = 0.722, p < 0.05, CI [0.080, 0.957]). Tumor size did not correlate with driving performance, nor did the other cognitive tests. Furthermore, there was no significant difference between patients with frontal tumors (n = 5) and parietal tumors (n = 5) in driving performance. Although patients reported having more emotional concerns than healthy controls (i.e. greater HADS scores), these symptoms did not correlate with driving errors.

**Table 6:** Correlation results between driving simulator errors (City Driving and Bus Following) and clinical variables, self-reported driving behavior, and cognitive test scores for participants with brain tumor.

	City Driving Total Errors	City Driving Speed Exceedances	Bus Following Total Errors	Bus Following Speed Exceedances
Months since diagnosis	**0.798** [0.462, 0.950]*	0.507 [−0.070, 0.801]	**0.840** [0.534, 0.951]**	**0.753** [0.357, 0.908]*
Months since surgery	0.279 [−0.412, 0.849]	−0.161 [−0.639, 0.429]	0.560 [−0.126, 0.905]	0.358 [−0.275, 0.811]
Tumor size (cm^3^)	0.468 [−0.103, 0.804]	0.190 [−0.415, 0.721]	0.218 [−0.387, 0.685]	−0.180 [−0.582, 0.452]
Self-report speed compared to traffic in city	−0.243 [−0.727, 0.447]	0.223 [−0.405, 0.677]	−0.339 [−0.744, 0.253]	−0.177 [−0.587, 0.284]
Self-report speed compared to traffic on highway	−0.043 [−0.636, 0.571]	0.357 [−0.164, 0.792]	−0.168 [−0.699, 0.449]	0.088 [−0.405, 0.567]
Self-report avoid driving in busy traffic	−0.382 [−0.830, 0.297]	**−0.685** [−0.817, −0.296]*	−0.454 [−0.826, 0.162]	−0.473 [−0.825, 0.108]
Self-report drive faster than speed limit if driver thinks he/she will not be caught	0.392 [−0.197, 0.758]	0.313 [−0.239, 0.772]	−0.035 [−0.590, 0.610]	0.300 [−0.299, 0.841]
UFOV Processing Speed	−0.290 [−0.723, 0]	−0.116 [−0.532, 0.239]	−0.060 −0.520, 0.332]	−0.315 [−0.661, 0.168]
UFOV Divided Attention	0.216 [−0.532, 0.817]	0.359 [−0.363, 0.833]	0.348 [−0.239, 0.833]	0.445 [−0.408, 0.913]
UFOV Selective Attention	0.523 [−0.171, 0.774]	0.222 [−0.425, 0.753]	0.**722** [0.080, 0.957]*	0.535 [−0.094, 0.934]
TMT-A time	0.122 [−0.562, 0.866]	−0.331 [−0.755, 0.317]	0.291 [−0.371, 0.855]	−0.093 [−0.672, 0.627]
TMT-A errors	0.466 [0.466, 0.746]	0.387 [0.234, 0.741]	0.314 [0.079, 0.674]	0.164 [−0.120, 0.582]
TMT-B time	0.177 [−0.437, 0.635]	−0.135 [−0.662, 0.481]	0.402 [−0.330, 0.862]	0.023 [−0.542, 0.711]
TMT-B errors	0.236 [−0.415, 0.759]	0.219 [−0.320, 0.663]	0.125 [−0.332, 0.571]	0.160 [−0.374, 0.669]
Digit span forward	0.196 [−0.438, 0.699]	0.372 [−0.288, 0.856]	0.232 [−0.377, 0.706]	0.461 [−0.048, 0.764]
Digit span backward	−0.018 [−0.565, 0.646]	0.060 [−0.523, 0.602]	−0.126 [−0.694, 0.477]	0.013 [−0.553, 0.569]
Digit symbol time	0.136 [−0.545, 0.741]	0.033 [−0.616, 0.636]	0.089 [−0.590, 0.826]	−0.227 [−0.762, 0.449]
Verbal fluency total correct	0.473 [−0.301, 0.830]	0.499 [−0.170, 0.891]	0.494 [−0.108, 0.806]	0.630 [−0.038, 0.890]
Verbal fluency perseverations	0.373 [−0.259, 0.850]	0.365 [−0.296, 0.882]	0.274 [−0.270, 0.701]	0.455 [−0.077, 0.824]
Verbal fluency switches	0.456 [−0.280, 0.831]	0.531 [−0.161, 0.897]	0.473 [−0.161, 0.845]	0.569 [−0.014, 0.862]
HADS anxiety	0.432 [−0.127, 0.798]	0.155 [−0.469, 0.659]	0.339 [−0.327, 0.783]	0.505 [−0.135, 0.892]
HADS depression	−0.134 [−0.748, 0.722]	−0.392 [−0.855, 0.332]	−0.132 [−0.626, 0.401]	−0.173 [−0.706, 0.551]
HADS total score	0.326 [−0.337, 0.791]	0.055 [−0.587, 0.617]	0.283 [−0.363, 0.664]	0.344 [−0.283, 0.792]

Note: All values are reported in Spearman correlation coefficient [95% confidence interval] format. HADS, Hospital Anxiety and Depression Scale; TMT-A, Trail Making Test Part A; TMT-B, Trail Making Test Part B; UFOV, Useful Field of View test. City Driving total errors are the sum of: collisions, speed exceedances, centerline crossings, road edge excursions, stop signs missed. Bus Following total errors is the sum of: speed exceedances, centerline crossings, road edge excursions. *p < 0.05, **p < 0.001.

## Discussion

This study investigated the self-reported driving habits and simulated driving performance of patients with brain tumors. To our knowledge, this is the first study to empirically assess the driving performance of patients with brain tumors using driving simulation technology.

Results from the self-reported questionnaire show that generally, patients did not report deficits in driving performance and less than 20% of patients self-restricted their driving after their diagnosis. Patients reported experiencing only little difficulty with driving in the rain or snow, at night or in high-traffic. Hence, these patients are not only driving after a brain tumor diagnosis, they are willingly engaging in potentially challenging and high-risk driving environments. Despite the confidence in their driving abilities, almost 40% sustained at least one collision since their tumor diagnosis, which may illustrate a discrepancy between self-reported driving ability and on-road performance. Lastly, 10–20% of patients reported engaging in risky driving behavior such as driving faster than the speed limit and engaging in angry behavior on the road.

The driving simulation found that patients with brain tumors did not exhibit global impairment (i.e. total number of errors), increased risk of collision, or impairment in lane control. Despite this, however, patients did exhibit impairments across two measures of speed control: speed exceedances and speed variability. Difficulty with speed control is an important factor in roadway safety, as it has been shown to be a risk factor for collision and severity of collision^11^. Both absolute speed and speed variance relate positively to crash rate^11^. Hence, although the patients in this cohort did not demonstrate a higher collision rate, their greater speed exceedances and variability put them at a greater risk for critical on-road events.

Our data suggest that patients who have undergone surgery for brain tumor resection are at risk of impaired speed control, yet may not be aware of this deficit, as over 70% of the questionnaire respondents indicated they drive at the same speed as other drivers in the city and on the highway. This finding is important, as it demonstrates that patients with brain tumors may lack awareness of their on-road behaviors, which is consistent with the finding that executive function is a common cognitive domain affected in these patients^12^. Clinically, it suggests that physicians may not be able to rely on self-reported changes in driving habits and behaviors; thus, determination of fitness to drive would benefit from collateral information from caregivers and possibly a more formal assessment by a specialist.

There is a wider literature on driving impairments in patients with Alzheimer’s disease (AD), mild cognitive impairment (MCI) and stroke, and the various predictive markers of driving impairment. A recent systematic review and meta-analysis of driving studies in AD and MCI found that measures of executive function, attention, visual-spatial ability and global cognition are the most predictive of driving performance, with effect sizes >0.5^13^. They also found that the TMT (marker of attention, processing speed and mental flexibility) and Maze Task (measure of planning and visual spatial ability) were the best single predictors of driving performance in patients with AD and MCI. These cognitive tests predict on-road performance more accurately than with driving simulation. Similarly, a study of driving simulation in patients with MCI showed that the TMT and UFOV Processing Speed were significantly associated with total driving errors^7^. In the stroke population, the measures most predictive of driving performance included TMT, UFOV and the Rey-Osterreith Complex Figure design^7,14^.

In our study, the only predictive cognitive measure was the UFOV Selective Attention; TMT did not significantly predict overall driving performance or specific driving errors. This may be attributed to the absence of cut-off scores for these cognitive tests, small sample sizes in the literature, heterogeneous cognitive deficits and disease progression in patients with various neurological conditions, amongst many others. Hence, it is possible that the executive function tests explored in our study might not be the most appropriate for this clinical population and may not map sufficiently well onto the neural substrates required for this group to drive safely.

These findings call for more comprehensive and specific cognitive assessments for patients with brain tumors. It has been suggested that composite batteries may be more useful^15^, given the complex, multi-faceted nature of driving. Moreover, research in the field of driving impairments in stroke have alluded to various conceptual models of driving, including the dynamic model by Galski and colleagues^16^, where various factors are accounted for in driving performance. In a systematic review of driving impairments in stroke, Marshall *et al*. argued that components of various frameworks are needed to devise a comprehensive driving guide^14^. A new assessment model is needed that is more dynamic in nature to capture the integration of various cognitive processes that are required in real-world driving.

Current research on driving in patients with brain tumors has focused primarily on the physician’s responsibility to report unfit drivers. Studies have shown that driving advice given by treating clinicians is highly inconsistent^17–19^, largely reflecting the lack of accurate and empirically validated driving guidelines for this patient population. To date, the most detailed guidelines are those provided by the driving authorities in the United Kingdom, where recommendations are made even for histological subtypes of brain tumors^7^. Interestingly, to date there are no investigations on driving performance in patients with brain tumors to inform these guidelines and to devise histology-specific driving profiles. Unfortunately, our sample size was too small to conduct statistical analyses on the implications of histological subtype on cognitive testing and driving performance. Other research in the field has shown that advanced intracranial disease is not only associated with increased tumor burden, increased utilization of adjuvant therapies such as radiation therapy, poorer cognition, and lower capacity for reasoning/decision-making, but also increased utilization of complex narcotic regimens^20,21^. The use of a new narcotic is associated with increased on-road collisions and traffic violations and for that reason amongst several others, many palliative care doctors would consider this subset of the neuro-oncological population at greater risk of unsafe driving^20^.

This study represents the first attempt to characterize the driving performance of a sample of patients with brain tumors; however, there are few methodological limitations. Although we investigated patients with resected tumors who are seizure free and have driven at least once since their surgery, heterogeneity was present in terms of tumor characteristics (i.e. histology, location, size), time since diagnosis, and types of treatments undertaken; hence, the role of these characteristics could not be investigated in this current study. We do recognize that it is important to study such factors as histological subtype and their implications on fitness to drive. Hence, replication with a larger clinical sample is needed to arise to more generalizable conclusions that can inform policy and clinical decisions. Secondly, the subjective driving experience reported by patients was not corroborated by caregiver responses. Interestingly, our data shows that vision complaints were the second most common initial physical complaint, after seizures. This statistic largely reflects the representation of pituitary tumors in our dataset. All patients with pituitary tumors were back to normal vision at the time of driving renewal. Fourthly, some may suggest that a driving simulation task is not as realistic as real-world driving. Despite this, research has shown that driving simulator tasks are valid and correlated with on-road performance^22–25^. Although the number of speed errors may be higher on-road than during simulation^22^, research has generally supported the measure of speed control on the driving simulator^22–24^.

Our study is the first to provide empirical research on the driving habits and simulated driving behaviors of patients with brain tumors. Notably, it investigated the driving performance of patients who are seizure-free, which is the major driving restriction in neuro-oncological populations. The results from our study show that patients may unknowingly engage in unsafe driving behavior that puts them at higher risk for on-road errors. Specifically, they are making more speed exceedances that have been shown to increase the risk of on-road collision and severity of collisions. Identification of these at-risk patients is challenging since guidelines are inconsistent and patients may lack awareness of their driving performance, making self-reported driving habits unreliable in the clinical setting. Certain cognitive markers such as the TMT and Maze task are widely utilized in the driving literature and have the largest effect size in predicting driving performance in patients with AD and MCI^13^. In our study, the UFOV showed some utility in predicting errors in driving scenarios requiring a high degree of sustained attention, error detection and visual-motor control. Although patients in our cohort reported significantly more emotional symptoms than healthy controls, these did not emerge as predictive markers for driving simulation performance. Ultimately, driving is a complex task that involves the recruitment of a wide neural network^8^ that may be implicated in patients with brain tumors. No single test (cognitive or simulation-based) can predict driving performance with adequate sensitivity and specificity. This calls for either a composite battery of tests or more ideally, a more tailored driving assessment that is dynamic and matches the integration of various cognitive functions. More empirical research is needed to identify neural substrates of driving performance in this population and the factors that contribute to dangerous driving in order to help physicians accurately determine fitness to drive in patients with brain tumors.

## Electronic supplementary material

Supplementary Dataset 2

Supplementary Dataset 1

Supplementary Dataset 3
